# Identification of RNA Polymerase III-Transcribed SINEs at Single-Locus Resolution from RNA Sequencing Data

**DOI:** 10.3390/ncrna3010015

**Published:** 2017-03-21

**Authors:** Davide Carnevali, Giorgio Dieci

**Affiliations:** Department of Chemistry, Life Sciences and Environmental Sustainability, University of Parma, 43124 Parma, Italy; giorgio.dieci@unipr.it

**Keywords:** SINE, Alu, mammalian-wide interspersed repeats (MIR), RNA polymerase III, non-coding RNA, RNA-Seq, bioinformatics

## Abstract

Short Interspersed Element (SINE) retrotransposons are one of the most abundant DNA repeat elements in the human genome. They have been found to impact the expression of protein-coding genes, but the possible roles in cell physiology of their noncoding RNAs, generated by RNA polymerase (Pol) III, are just starting to be elucidated. For this reason, Short Interspersed Element (SINE) expression profiling is becoming mandatory to obtain a comprehensive picture of their regulatory roles. However, their repeated nature and frequent location within Pol II-transcribed genes represent a serious obstacle to the identification and quantification of genuine, Pol III-derived SINE transcripts at single-locus resolution on a genomic scale. Among the recent Next Generation Sequencing technologies, only RNA sequencing (RNA-Seq) holds the potential to solve these issues, even though both technical and biological matters need to be taken into account. A bioinformatic pipeline has been recently set up that, by exploiting RNA-seq features and knowledge of SINE transcription mechanisms, allows for easy identification and profiling of transcriptionally active genomic loci which are a source of genuine Pol III SINE transcripts.

## 1. Introduction

SINEs (Short Interspersed Elements) are retrotransposons of size ranging between 70 and500 bp, that invade eukaryotic genomes and, with more than 1.7 × 10^6^ copies, build up roughly 13% of the human genome [1]. Here, this group of retrotransposons comprises two main families of repeated elements: Alu, ancestrally derived from 7SL RNA [2], and MIR (Mammalian-wide Interspersed Repeat), which may have arisen following the fusion of a tRNA molecule and a 3’-end of an existing Long Interspersed Eelement (LINE) [3]. Both Alu and MIR are transcribed by RNA polymerase (Pol) III. Another, less numerous family of retrotransposons, SVA (SINE/VNTR/Alu), is sometimes classified as SINE, although it appears to be transcribed by Pol II instead of Pol III [4]. SINEs are capable of amplification in the hosting genome through a ‘copy and paste’ mechanism, which involves an RNA intermediate, and are considered non-autonomous since they need proteins encoded by LINEs to complete their retrotransposition cycle. Among SINEs, only Alus are currently active in their amplification process while MIRs ceased to amplify ~130 Myr ago [5,6]. Although SINEs are one of the most abundant elements in the human genome, their Pol III-driven expression levels are usually very low in normal conditions despite the high efficiency with which Pol III generally transcribes its target genes (e.g., tRNA genes). Indeed, SINE transcription is thought to be silenced by the cell through epigenetic mechanisms, mainly involving DNA and histone methylation [7,8]. Epigenetic silencing is likely essential to control and mitigate SINE spreading into the hosting genome which would rapidly lead to cell lethality because of their insertional mutagenic and genomic instability-causing potential. SINE expression has been shown to vary in response to different types of signals. However, the mechanisms of SINE expression regulation, which is likely to mainly occur at the transcriptional level, still need to be elucidated [9]. Historically considered as junk DNA, SINEs have been found to act as promoters, insulators, silencers and enhancers toward the expression of protein coding genes. They are thus now thought to represent important, yet largely unexplored elements of gene regulatory networks [10,11,12,13]. These roles, together with the recognized ability of SINE non-coding RNA (ncRNA) to regulate transcription and translation in *trans* [14,15], make it mandatory to further investigate their expression, especially at the resolution of individual SINE loci.

## 2. SINE Element Structure

**Figure 1:**
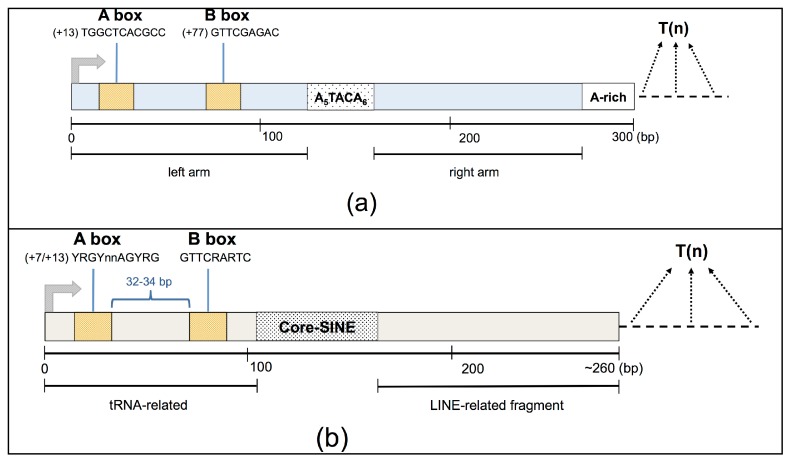
Short Interspersed Element (SINE) structures. (**a**) Representation of the structure of an Alu element of ~300 bp. Two monomers ancestrally derived from the 7SL RNA are separated from each other by an A-rich region with consensus sequence A_5_TACA_6_. Pol III transcription is driven by A- and B-box promoter elements located in the left arm which, together, form the binding site for TFIIIC. Another A-rich tract is located at the 3’ of the right arm, at the end of the *Alu* body. Pol III transcription is expected to terminate at the first encountered termination signal (T*n*) which may be located at varying distances from the end of the *Alu* body, thus allowing for the generation of Alu RNAs having 3’ tails of different lengths and sequences. (**b**) Representation of the structure of a mammalian-wide interspersed repeat (MIR) of ~260 bp. A tRNA-related region, containing A- and B-box promoter elements and a Long Interspersed Repeat (LINE-related fragment are separated by Core-SINE, a highly conserved central sequence. The unique genomic region downstream of the 3’ end of the MIR element (dashed line) could contain the Pol III termination signal (T*n*) which may be located at varying distances from the end of the MIR body. Adapted from [18,19].

Alu and MIR elements display a similar structure, composed of two monomers separated by an intervening DNA region. In particular, the body of a typical Alu element is about 300 bases in length, and is formed from two diverged, 7SL-related monomers separated by a short A-rich region, with a longer A-rich region located at the 3’ end of the element (Figure 1A). The complete MIR element is about 260 bp in length and possesses a tRNA-related 5’ head, a 70-bp conserved central domain containing a 15-bp core sequence, and a LINE-related sequence located at the 3’-end [3] (Figure 1B).

SINEs are potential targets of the RNA Pol III transcription machinery thanks to the presence of an internal bipartite promoter element located in their left monomer, which is composed of an A and a B box [16]. Transcription initiates at the beginning of the SINE and terminates at the closest poly(dT) termination sequence encountered downstream of the SINE body, generally in a SINE-unrelated genomic region of unique sequence. Thus, each genuine Pol III-derived SINE RNA has a well-defined 5’-end, usually located 12–15 bp upstream of the A-box promoter element, whereas its length can vary, depending on the location of the Pol III termination signal, being able to reach a maximum of ~600 bp [17].

Moreover, due to sequence mutations accumulated over time, many SINEs annotated in the human genome represent fragments of their corresponding ancestor full-length element that could also be a target of the RNA Pol III transcription machinery. In particular, both left and right SINE fragments could be a source of Pol III-derived chimeric transcripts originating from an upstream or ending in a downstream unique SINE-unrelated moiety. Indeed, while left SINE fragments contain Pol III promoters, right ones could still potentially be a target of the Pol III machinery complex if their upstream SINE-unrelated regions provide A- and B-box sequences that, for example, have been preserved from the mutation of an ancestral (now degenerated) SINE portion [18,19].

## 3. SINE Transcript Identification and Quantification: Techniques and Problems

Prior to the development and use of our bioinformatic pipeline SINE element, expression profiling has never been carried out in depth, because the identification of genuine Pol III-transcribed *Alu* and MIR RNAs is made difficult by (i) the extremely high copy number and sequence similarity of *Alu* and MIR elements within the human genome; and (ii) their frequent location within introns or untranslated regions of primary or mature Pol II transcripts.

To date, while no studies have been conducted on MIR expression profiling on a genome-wide scale, those aimed at identifying Pol III-derived *Alu* RNAs have been performed using low throughput techniques such as Northern hybridization [20], allowing to distinguish them from *Alu* RNAs passengers of longer Pol II transcripts, and size fractionation combined with C-RACE, followed by sequencing of the unique 3’ ends to identify the source loci of transcription [21]. Even if Northern hybridization is effective in global *Alu* RNA quantification, it fails in assessing the expression level of individual *Alu* loci, while techniques used to identify transcriptionally active *Alu* elements are unfeasible on a genomic high throughput scale. More recently, the development of Next-generation Sequencing (NGS) techniques has been exploited, through the use of Chromatin Immuno-Precipitation followed by massive parallel sequencing (ChIP-seq), to identify transcriptionally active SINE loci, whose association with components of the Pol III machinery has been used as evidence of their transcription, confirmed through other techniques for previously unknown selected loci [22,23,24]. However, even if this high-throughput technique has the advantage of being carried out on a genomic scale, the association of Pol III transcription factors (TFs) to a SINE element does not necessarily indicate its transcription. Quantification of expression levels is also not easy through this ChIP-based approach. Therefore, none of the above-mentioned approaches could be used for a comprehensive and quantitative expression profiling of SINEs.

The advent of RNA sequencing (RNA-Seq) for transcriptome profiling has overcome the limitations of previous methods, being able to allow both the identification of active transcription events and the source loci of Pol III-generated SINE RNAs [18] as well as estimating their abundance. However, with it being theoretically possible that SINE expressed elements identified by RNA-seq could result from an as-yet unidentified artifact of RNA-Seq, a combined approach which involves both RNA-seq and ChIP-seq techniques would strengthen their identification. Moreover, to perform such a thorough expression profiling of SINE elements, a number of technical and biological issues have to be taken into account to fully exploit the potential of RNA-Seq.

The first issue concerns the genomic position of SINE elements, which are frequently hosted by longer Pol II-transcribed genes, either within introns or within untranslated regions. It is likely that both canonical and pervasive Pol II transcription will generate transcripts containing an embedded SINE sequence, given SINE ubiquity throughout the human genome. Therefore, simply counting RNA-seq reads mapping to SINEs is not evidence of genuine Pol III-derived transcripts, but rather leads mainly to the detection of passenger SINE-containing Pol II transcripts. This is a key concept that must be carefully taken into account to avoid attributing RNA-seq reads to transcriptionally autonomous, Pol III-dependent SINEs, which should be better attributed to host Pol II transcripts. Overlooking this problem could lead, for example, to a regulatory change in the expression of host, Pol II-transcribed protein-coding or long non-coding RNA (lncRNA) genes being mistakenly attributed to differential SINE expression. A further difficulty to be taken into account for true SINE RNA profiling, as discussed above, is the relationship between the length of a Pol III-derived SINE RNA and its genomic source locus, which is not as well defined as for Pol II transcripts, with it being possible for the Pol III transcription machinery to transcribe genomic regions both upstream and downstream of the annotated element. Therefore, in summary, just counting reads on the annotated SINE element does not give a complete picture of their genuine activity as Pol III transcription units.

Another issue to be noted, arising from the repetitive nature of SINE elements, is the problem of reads mapping to multiple locations onto the genome (multireads). To address such an issue in RNA-seq data analysis, three main possibilities have been recognized and devised: the so-called ‘unique’, ‘best match’ and ‘all matches’ strategies (reviewed in [25]). With respect to this, a frequently overlooked feature of SINEs is that only rarely are they perfectly identical throughout their whole length, given sequence mutations accumulated over time [26]. This issue could thus be mitigated using sufficiently long or paired-end RNA-seq reads so that the ‘unique’ alignment strategy could be successfully exploited for the identification of Pol III-derived SINE transcripts at single-locus resolution.

Strictly correlated with the multimapping read problem is the quantification issue. Recently, a method has been proposed to estimate the transcriptional enrichment of repetitive elements from NGS data by applying strategies for the allocation of multimapping reads [27]. However, this method can only assign multireads to the family and subfamily of the repeat element from which the reads arise, allowing accurate enrichment estimates only at these levels. Thus, with it being impossible to assign a multimapping read exactly to its source, it follows that expression quantification for SINE elements at single-locus resolution can only be done using uniquely mapped reads. This strategy could produce a bias, the magnitude of which depends on the fraction of multireads, when comparing expression levels of SINE elements against those of other genes not containing repetitive sequences; however, it will not represent a problem as long as we are comparing SINE expression levels among different cell types or conditions which have been sequenced using the same protocol. Despite the fact that discarding multimapping reads could affect the quantification of repeat elements, especially those consisting mainly of non-unique sequences, using sufficiently long paired end reads would affect SINEs expression quantification only minimally. Indeed, we verified that the fraction of multireads on SINE elements in the human genome (*Alus* and MIRs) are as little as 2% when using 75-nt-long paired-end stranded reads from poly(A)− RNA of GM12878 cells from ENCODE CSHL Long RNA-Seq data (data not shown) [18]. Therefore, using sufficiently long or paired end reads, the uniquely mapped ones would allow a satisfactory quantification of the expression levels of each individual SINE in the genome.

Considering all the above issues, and the importance of solid strategies for SINE expression profiling to shed new light on their regulatory potentials, we developed a bioinformatic pipeline that exploits the key feature of SINE element architecture and their transcription mechanisms, along with RNA-Seq technical features, to identify genuine Pol III-derived SINE RNAs at single-locus resolution.

## 4. A Bioinformatic Pipeline for SINE Expression Profiling

First and foremost, it must be considered that Pol III transcripts are not polyadenylated and therefore poly(A)− RNA should be used for their identification. However, since *Alu* RNAs usually possess an A-rich tail and could be captured using a poly(A)+ RNA extraction protocol [18], the use of total RNA is recommended. To mitigate the multimapping issue, we suggest using at least 75-nt stranded paired-end sequencing reads, which additionally give the opportunity to discover genuine SINE transcription units overlapping with Pol II genes but in an antisense orientation. Moreover, strandedness restricts the number of degrees of freedom during the mapping process, giving the spliced aligner more chances to identify the unique source of the paired-end reads.

**Figure 2:**
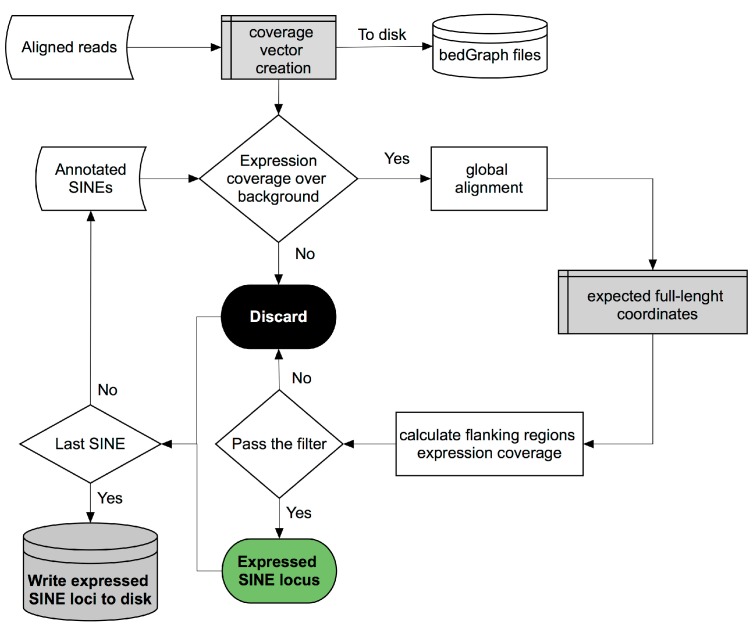
Bioinformatic pipeline for SINE RNA expression analysis at single locus resolution. Shown is a flow-diagram of a recently developed bioinformatic pipeline for the identification of autonomously expressed SINE loci from RNA-seq data sets [18,19]. See text for details.

Our bioinformatics pipeline [19] is outlined in Figure 2, and consists of three main steps: (1) genome expression coverage vector creation; (2) global alignment between the annotated element and its corresponding full-length sequence; (3) application of a filter aimed at excluding passenger SINE transcripts.

**Figure 3:**
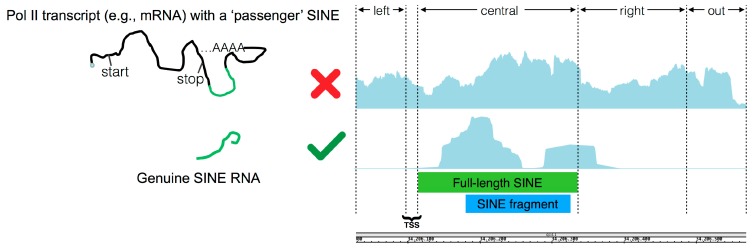
Graphical representation of the Flanking Region Filter. By comparing the expression coverage signals of the regions flanking the SINE body, the filtering step of our bioinformatics pipeline allows to discriminate between genuine Pol III-derived SINE RNAs and those hosted in longer Pol II transcripts (e.g., in their 3’ UTRs).

The first step in the pipeline creates a vector representing the genome expression coverage from uniquely mapped reads from the supplied *bam* file and calculates the value of the background noise signal by dividing the total expression coverage by human genome size, that is 3 or 6 billion nucleotides, depending on whether unstranded or stranded sequencing reads are used, respectively. After genome expression coverage calculation, the pipeline iterates over each annotated SINE element, supplied in *GTF* format (General Transfer Format), to check if its expression coverage exceeds the pre-calculated background noise by *n* times, which is a settable value. If this is the case, given the fact that many annotated SINE elements represent incomplete fragments of their corresponding full-length element, a global alignment using the Needleman–Wunsch algorithm between the annotated element and the corresponding full-length consensus sequence is performed in order to obtain the boundary genomic coordinates of the potentially ancestral full-length element, named ‘central body’, and to define the coordinates of the flanking regions (‘left’, ‘right’ and ‘out’) necessary to apply the final filter. The ‘right’ region corresponds to that immediately downstream of the end of the full-length SINE element which could contain the poly(dT) Pol III termination signal. Its length has been set by default to 200 bp given the average length of full-length SINE of ~260 bp and with Pol III being potentially able to generate a 600 bp transcript [17]. ‘Left’ and ‘out’ regions, instead, represent the upstream and downstream regions which are not part of the SINE transcript and therefore not involved in Pol III transcription. The last step in the pipeline consists of a filtering process, named ‘Flanking Region Filter’, aimed at distinguishing genuine Pol III-derived SINE RNAs from those passengers of longer Pol II transcripts. The filter works by verifying whether the expression coverage in the ‘central body’ is highly enriched with respect to that in the pre-calculated upstream ‘left region’ and downstream ‘out region’, and higher than the ‘right region’ given the fact that Pol III transcription termination should occur in this region at any distance from the end of the full-length SINE element (Figure 3).

The speed of the bioinformatic pipeline mainly depends on two factors: (1) the number of mapped reads in the *bam* file; and (2) the number of SINE elements having an expression coverage fold enrichment over the background noise signal, which could be adjusted through the *‘-bg*’ parameter. The computational time for expression coverage vector creation can be avoided in subsequent runs by supplying the *Bedgraph* coverage vector file, converted to the *BigWig* format, created in the first run, to the pipeline. Table 1 summarizes the speed of the pipeline using a ~200 million mapped reads *bam* file, two different values for SINE coverage expression enrichment and two different numbers of supplied SINE elements to be analysed.

To test the precision and recall of our pipeline, we used Flux Simulator [28] to generate 76 nt-long paired-end reads from the simulated expression levels of 331 genuine Pol III SINEs and both protein-coding and non-coding RNAs (lincRNA and pseudogenes) along with lower expression levels of their introns to simulate a background noise signal. Table 2 reports the precision and recall values for two runs having different ‘*-bg*’ values which affect both the total number of discovered genuine Pol III SINEs as well as the true positives. The pipeline provides other parameters that could be used to adjust its sensitivity depending on the type of RNA-seq data available (e.g., read length, single or paired-end, strandedness) and the genomic location of the SINEs of interest (e.g., intergenic or intragenic).

Speed, precision and recall computational analyses have been performed using one CPU and 16 Gb memory of a dual-twelve-core 2.2 GHz Intel ES-2650v4 CPUs.

## 5. Conclusions

To date, the identification of genuine Pol III-derived SINE transcripts performed either using low throughput techniques or ChIP-Seq approaches could not give a satisfactory and comprehensive picture, with it not being possible to simultaneously quantify and identify them at single-locus resolution. On the other hand, the RNA-Seq approach, which has the potential to overcome the limitations of previous methods, has only been used for the enrichment estimation of repetitive elements at the family and subfamily level. Moreover, given the high frequency with which SINE elements are located within longer Pol II-transcribed genes (e.g., protein-coding genes), attempts to identify their expression by simply counting RNA-Seq reads mapping onto them does not solve the issue of distinguishing ncRNAs derived from autonomous Pol III transcription units from longer hosting Pol II transcripts. This ambiguity generates biases and data misinterpretation when performing differential expression analyses. The recently developed pipeline discussed above aims to overcome these issues by exploiting RNA-Seq technology along with the peculiar characteristics of Pol III SINE RNAs, thus allowing, for the first time, to profile SINE expression at single-locus resolution [18,28]. The availability of such key information, used along with other genomic and epigenomic data, opens new scenarios for the study of SINE transcription regulation—an important yet far too unexplored field given their relevance for human genome stability, expression and evolution.

## Figures and Tables

**Table 1:** Bioinformatic pipeline speed evaluation. The table shows the speeds of our bioinformatic pipeline for four different runs on a *bam* file having ~200 million 76 nt-long stranded RNA-seq paired-end reads aligned to the human genome (GRCh37). The number of SINEs having an expression coverage over the calculated background signal (column 3) arises from different ‘*-bg*’ parameters in column 1 (see text for details). The step in the pipeline for expression coverage vector creation from the supplied *bam* file took almost 2 h.

-bg	SINEs Analysed	SINEs over Background	Identified Genuine Pol-III SINEs	Total Run Time (h)
3	430 k	2872	42	2.6
2	430 k	4576	119	2.8
3	1100 k	44,973	175	6.9
2	1100 k	64,425	463	10.9

**Table 2:** Bioinformatic pipeline precision and recall. The table shows the precision and recall for two different runs on a ~10 Gb *bam* file with ~200 million reads mapped onto the human genome (GRCh37) containing the simulated expression of 331 genuine Pol III SINEs Column 1 reports the expression coverage fold change enrichment used (see text for details).

-bg	Genuine Pol III SINEs Found	True Positive	Precision	Recall
2	222	210	0.95	0.63
3	195	195	1	0.59
